# Effects of Physical Exercise and the use of Doxorubicin on Cardiac Function in Rodents: A Systematic Review and Meta-Analysis

**DOI:** 10.2174/011573403X328856241219114652

**Published:** 2025-02-10

**Authors:** Bruno Gama Linhares, Diego Gama Linhares, Rodrigo Gomes de Souza Vale

**Affiliations:** 1 Research Center in Physical Activity, Health, and Leisure (CIAFEL), Faculty of Sport, University of Porto, Porto, Portugal;; 2 Health Sciences Laboratory, Estácio de Sá University, Campos dos Goytacazes, Rio de Janeiro, Brazil;; 3 Laboratory of Exercise and Sport, Institute of Physical Education and Sports, Rio de Janeiro State University, Rio de Janeiro, Brazil

**Keywords:** Chemotherapy, cancer, quality of life, cardiotoxicity, doxorubicin, fractional shortening

## Abstract

**Background:**

Anthracycline-based chemotherapy, such as Doxorubicin (DOX), often induces cardiotoxicity in cancer patients, which compromises their health and quality of life.

**Objective:**

This study aimed to verify the effects of exercise concomitant with prolonged administration of DOX on improving cardiotoxicity.

**Methods:**

A systematic literature search in MedLine, PubMed, Web of Science, and Scopus databases was performed from inception until November 2023, strictly following the Preferred Reporting Items for Systematic reviews and Meta-Analyses (PRISMA) statement. Preclinical randomized controlled trials related to exercise, cardiotoxicity, and DOX were included.

**Results:**

Eight studies were included in the systematic review and 7 were selected for meta-analysis. By evaluating the Fractional Shortening (FS), it was possible to verify that exercise as complementary therapy provided a cardioprotective effect when compared to DOX combined with sedentary behavior (heterogeneity: I^2^ = 53%; tau^2^ = 0.19; *p =* 0.03; overall effect: z = 2.69; *p <* 0.01). However, no additional benefits were observed for the Left Ventricular Ejection Fraction (LVEF) (heterogeneity: I^2^ = 82%; tau^2^ = 1.43; *p <* 0.01; overall effect: z = 1.42; *p =* 0.15).

**Conclusion:**

The included studies demonstrated exercise to have a cardioprotective effect on rodents, mainly on FS. However, there is a lack of high-level evidence to guide exercise prescription in clinical practice to improve cardiotoxicity associated with DOX administration.

## 1 INTRODUCTION

Anthracycline-based chemotherapy, such as Doxorubicin (DOX), often induces cardiotoxicity during its administration [1-3]. Doxorubicin (DOX) continues to be a major constituent of many cancer treatment regimens [4-6]. Detection, improvement, and protection of cardiotoxicity are important due to the large number of patients treated with DOX, because, despite its efficiency, the cumulative toxicity related to its dose restricts the treatment [7]. The prevalence and severity of cardiotoxicity occur as a function of time and DOX dosage and may emerge either early or years later [8]. Cardiotoxicity-related symptoms can vary from arrhythmias, asymptomatic structural changes, or increased blood biomarkers, to urgent hospitalization [9].

The most frequent and clinically relevant form of cardiotoxicity is heart failure, identified by Left Ventricular Ejection Fraction (LVEF) (an index of cardiac pumping efficiency), which occurs in the first year and is associated with anthracycline dose [10, 11]. Left ventricular dysfunction, whether subclinical or fulminant, may preclude the continuation of chemotherapy. Therefore, it is important to identify these patients as soon as possible, thus minimizing heart damage and facilitating continued chemotherapy, usually evaluated by echocardiography [12-16]. Since the early 1950s, when Hertz and Edler described the use of ultrasound to assess mitral valve disease [17], echocardiography has been the most used non-invasive tool in cardiology to assess morphological and functional heart diseases [18]. This technique uses high-frequency ultrasound for heart visualization, providing relevant information on cardiac dimensions [19], particularly the left ventricle structural-related variables [20]. The Fractional Shortening (FS, an index of cardiac contractile performance) is the most used index for ventricular mechanics assessment [18, 21-23], and corresponds to the percentage change in the diameter of the left ventricle during ventricular systole [24, 25]. Both LVEF and FS are used to assess the function of the heart's left ventricle, the main pumping chamber of the heart. To calculate LVEF (typically expressed as %), end-diastolic and systolic volumes are necessary, which are complex assessments since the technology dependency can affect the reliability of the measurements, while for FS (expressed as %), left ventricular end-diastolic and systolic diameters are needed, which are easier assessments to perform [19-26]. The LVEF focuses on the volume of blood pumped out, while FS focuses on the change in diameter during the cardiac cycle. These metrics provide important evidence about the heart's ability to pump blood effectively, specifically in the heart disease context. Both pumping efficiency and contractile performance of the heart's left ventricle are important for clinicians to diagnose and monitor heart conditions [19-26].

Physical inactivity is a deleterious factor that worsens cancer outcomes, such as weakness, muscle fatigue, and death, through increased chronic systemic inflammation and oxidative stress [26]. Interventions, including exercise programs, as part of supportive cancer care, are low-cost and scalable, reducing the anthracycline-related damage to the heart, aorta, and heart muscle, *i.e*., cardiovascular health [27]. Although the role of exercise in improving DOX-induced cardiac dysfunction has been consistently demonstrated in pre-clinical models, many studies begin weeks or even months before an acute treatment (bolus administration of an accumulated dose of DOX) [28, 29]. Cytotoxic chemotherapy treatment usually starts as soon as possible after diagnosis, limiting the window of opportunity for preconditioning [30], *i.e.,* physical preconditioning seems not relevant for clinical practice.

Protocols combining acute doses of DOX with exercise for a short treatment period do not allow enough time for systolic function deterioration as expected with exercise concomitant with chronic doses (fractioned dose along time) of DOX administration. Likewise, it does not provide the cardiovascular benefits demonstrated by regular exercise programs [31, 32]. Exercise modulates some important cardiac defense systems to upset the toxic effects triggered by DOX administration [33-36], like antioxidant capacity (probably attributed to mitochondrial plasticity), as related adaptations can be translated into improved cardiac function in the cardiomyopathy context [37-39]. It is also suggested that exercise is a strategy to improve DOX toxicity (preconditioning) or to act as a complementary therapy during DOX administration [40]. Despite that, the benefits of exercise in improving cardiotoxicity associated with DOX administration are not yet clear in clinical studies. In a recent systematic review [41], due to the great heterogeneity among the extracted studies, the authors were unable to conclude that exercise is cardioprotective when comparing LVEF from cancer patients between control and exercise groups.

Most studies involving physical exercise programs as well as the use of DOX generally occur before DOX administration [42], suggesting greater benefits during this period [43], and reducing its annual mortality rate [44]. Despite the chemotherapy-adopted protocol, depending on the disease progression stage [45], the earlier the diagnosis and beginning of the treatment, the higher the survival rate [46]. Thus, there is often not enough time to apply exercise as a complementary intervention in clinical practice (before or after DOX-based chemotherapy), *i.e.,* in human research, as a delay in the DOX administration can impact the life of the patient. Instead, research on animals allows a deeper understanding of the effects of exercise as a complementary therapy at different timings intervention during DOX administration. Thus, this review aimed to verify if concomitant exercise as complementary therapy can offer cardioprotection during DOX administration.

## 2 METHODOLOGY

### 2.1 Study Design and Protocol Registration

This systematic review and meta-analysis is reported according to the Preferred Reporting Items for Systematic reviews and Meta-Analyses (PRISMA 2020) statement [47]. The study protocol is registered in the International Prospective Register of Systematic Reviews (PROSPERO; www.crd.york.ac.uk/prospero/) section database (number: CRD42022366657).

### 2.2 Eligibility Criteria

In this study, eligible pre-clinical Randomized Controlled Trials (RCTs) published as full-text manuscripts in any language were included. Eligibility criteria comprised indexing terms from PICOS approach components (Participants, Interventions, Comparators, Outcomes, and Study design) as follows:

- Participants: Sedentary rodents medicated with DOX for 1 week or more, developing cardiotoxicity, as confirmed by echocardiography, without associated comorbidities or in concomitant treatment with other drugs and supplements.
- Intervention: Aerobic exercise protocols concomitant with DOX administration. We included studies with rodents that used all types of DOX administration (greater than 3 applications) aiming to induce cardiotoxicity. Under the same dosage conditions, we could compare the control group with the group that underwent the intervention through exercise.
- Comparators: Rodents that performed aerobic exercise protocols (exercise group) compared with those that did not (control group).
- Outcomes: Evaluation of cardiac function through echocardiography using FS as the primary outcome.
- Study design: Randomized Controlled Trials (RCTs).

Exclusion criteria: Studies that exclusively addressed liver, muscle, or kidney toxicity, cardiotoxicity caused by the administration of a single (acute) or more doses of DOX before or after the beginning of the exercise program and studies that exclusively involved physical preconditioning, hormonal modulation in DOX-administrated animals, comorbidities (diabetes, hypertension, or mental preconditions), or interventions combined with exercise (restricted diet or supplementation), were excluded.

### 2.3 Search Strategy

Two independent authors conducted systematic web-based literature searches across Medline, PubMed, Web of Science, and Scopus platforms from inception through March 2024. The search strategy included Medical Subject Headings (MeSH) terms related to exercise, cardiotoxicity, DOX, and animals. We selected studies that evaluated the impact of concomitant exercise on cardioprotection or mitigation of cardiotoxicity caused by DOX administration. There were no limits on language or publication status. The full search strategy is shown in Appendix **1**.

### 2.4 Study Selection

The results of the searches in the databases were combined into an Endnote X9 software file [48]. A two-step study selection involved the evaluation of titles and abstracts by two independent reviewers, followed by the full-text assessment of all potentially eligible studies. In case of disagreement, a third reviewer was consulted to determine the eligibility of the study.

### 2.5 Data Extraction

The studies were summarized by entering data in a spreadsheet, including information about the authors, year of publication, the study population (animal breed, sex, age, weight), study characteristics (number of animals, duration, and time of intervention), characteristics of anthracycline administration (time, dose, and number of doses), exercise protocol (modality, frequency, duration, and intensity), and cardiac function assessment methodologies. The main outcome referred particularly to the FS (value in %), which must have been assessed by echocardiography. If FS (mean ± SD) was not displayed numerically, we used WebPlotDigitizer (https://apps.automeris.io/wpd/) to extract data from the graphs of the selected studies. Data from some studies [49-52] were transformed from Standard Error (SE) into standard deviation (SD) using the formula below (equation 1):

SD=SE×√(sample size) (eq 1)

### 2.6 Methodological Quality Assessment

The methodological quality analyses were performed using three different tools: (1) Systematic Review Centre for Laboratory Animal Experimentation (SYRCLE) [49-53], (2) Collaborative Approach to Meta-Analysis and Review of Animal Data from Experimental Studies (CAMARADES) [54], and (3) Review Manager (RevMan software, version 5.4.1; The Nordic Cochrane Center, The Cochrane Collaboration, Copenhagen, Denmark). Both SYRCLE and CAMARADES specifically used for animal experimentation are at risk of bias.

SYRCLE has 10 items and uses “yes” (Y) for low risk of bias, “no” (N) for high risk of bias, or “unclear” (U) for insufficient detail to measure the risk of bias. CAMARADES is a tool with unfixed items that include sampling methods, blinding, and adequacy of the protocol according to the outcome of interest, a “present” (1) or “absent” (0) scoring system for each evaluation criterion, and an arithmetic sum that is done at the end. RevMan uses 7 items, involving “H” for high risk of bias, “L” for low risk of bias, or “U” for uncertain risk of bias, through colors red, green, or white, respectively, for each item.

### 2.7 Evidence-level Assessment

Two authors independently assessed the certainty of evidence using the Grading of Recommendations Assessment, Development, and Evaluation (GRADE) approach with the GRADE PRO website, available at https://gradepro.org. GRADE specifies four categories: “high”, “moderate”, “low”, and “very low”, applied to a body of evidence. RCTs begin with high-quality evidence. Five aspects can decrease the quality of evidence: methodological limitations, inconsistency, indirect evidence, inaccuracy, and publication bias. On the other hand, three aspects can increase the quality of the evidence: effect size, dose-response gradient, and confounding factor [55].

### 2.8 Statistical Analysis

A random-effects model was employed for each selected outcome. Pooled Effect Sizes (ES) were presented as Standard Mean Difference (SMD) with a 95% Confidence Interval (95%CI). Sensitivity analyses were conducted to detect if any study was responsible for a large proportion of heterogeneity (I2), which was assessed and qualitatively considered not important if I2 = 0–40%, moderate if I2 = 30–60%, substantial if I2 = 50–90%, and considerable if I2 = 75–100% [56]. Publication bias assessment was not performed because outcomes analyses included less than 10 studies [57]. The package “meta” (version 4.11–0) for the R statistical software (version 4.1.0) was used. Overall effects (z-value) were considered statistically significant at *p*-value < 0.05.

## 3 RESULTS

### 3.1 Study Selection and Characteristics

**Fig. (1):**
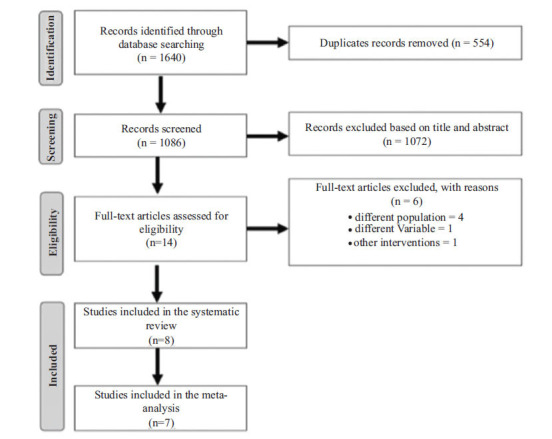
Flow diagram indicating the number of studies retrieved in the literature search, and the final number of studies included in the meta-analysis.

The initial search resulted in 1,670 studies. From these, 8 studies [49-52, 58-61] were included in the systematic review and 7 were selected for meta-analysis (Fig. **1**) [49-52, 58, 60, 61]. Regarding the excluded study [55], we were unable to obtain FS values.

Table **1** summarizes the sample and study characteristics. The 7 studies included in the meta-analysis used 307 rodents, 3 studies [51, 58, 61] used Sprague-Dawley rats (50.8%), and 4 studies [49, 50, 52, 60] used C57BL/6 mice (49.2%). From these, 81 animals were allocated to the Saline (SAL) + Sedentary (SED) group, 95 were allocated to the DOX + SED group, 89 were allocated to the Exercise (EXE) + DOX group, and 42 were allocated to the EXE + SAL group. Thus, 57.3% were allocated to the sedentary group and 42.7% to the trained group. Two studies [49, 51] used females (36.8%) and five [50, 52, 58, 60, 61] (63.2%) used males. Three studies reported the age of the animals at 8 weeks [49, 50, 52]. In two studies, the animals were 4 weeks old and reported as juveniles [58, 60]. Finally, two studies did not mention the age of the animals [51, 61].

The studies evaluated in this review used the cumulative value of DOX of 4 mg·kg^-1^ [52], 10 mg·kg^-1^ [58], 14 mg· kg^-1^ [59], 15 mg·kg^-1^ [51], 25 mg·kg-1 [50], 32 mg·kg^-1^ [49], and 300 mg·kg^-1^ [61]. The protocols for anthracycline-induced cardiomyopathy were highly heterogeneous regarding the fractioning of the cumulative dose, frequency of injections, and timing of administration concerning the exercise protocol.

Two studies [51, 58] used voluntary running (free voluntary access 24 h·day^-1^) and five [49, 50, 52, 60, 61] used motorized treadmills. Regarding the exercise programs proposed by the studies that composed our review, all used low/moderate intensity exercise programs, *i.e*., at ~40-60% of maximal oxygen uptake (〖”V” ˙”O” 〗_”2MAX”) [62].

### 3.2 Methodological Quality Assessment

The methodological quality was assessed through two risk-of-bias assessment tools: SYRCLE (Table **2**) and CAMARADES (Table **3**). Most of the studies included in this review were classified as uncertain or at high risk of bias according to allocation concealment or blinding of participants and staff, mainly due to a lack of description of blinded assessment of outcomes, monitoring of body weight variables, and sample size calculation. They did not report blinding of the investigators regarding the care and/or evaluation of the animals. Only one study [49] provided information on blinding of the examiner of the main outcome, and the presence of bias remained unclear for all studies as they all lacked this information.

### 3.3 Meta-analysis

#### 3.3.1 Effects of Exercise on FS and LVEF

**Fig. (3):**
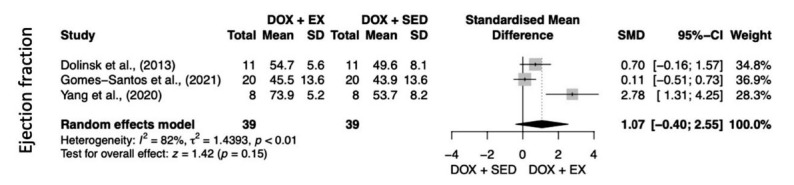
Left ventricular ejection fraction (LVEF) meta-analysis. Total: number of studies included in the analysis, SMD: standard mean difference, 95%CI: confidence interval, I2: heterogeneity, Z (p): test for overall effect and *p*-value. *Statistical significance: *p* ≤ 0.05. **Abbreviations:** DOX + EX: Doxorubicin exercise intervention group; DOX + SED: Doxorubicin control group.

Fig. (**2**) shows the analysis of the effects of exercise on FS. The DOX+EXE provided cardioprotective effect when compared to DOX+SED (heterogeneity: I^2^ = 53%; tau^2^ = 0.19; *p =* 0.03; overall effect: z = 2.69; *p <* 0.01), but without additional benefits for the LVEF (heterogeneity: I^2^ = 82%; tau^2^ = 1.43; *p <* 0.01; overall effect: z = 1.42; *p =* 0.15) (Fig. **3**).

Table **4** shows the level of evidence of the comparison between DOX+EXE *vs.* DOX+SED with respect to FS and LVEF. Regarding FS (9 RCTs; 240 animals), we observed high-level evidence of the pooled studies (SMD 0.55; 95%CI 0.15 to 0.95). Regarding the LVEF (3 RCTs; 78 animals), we observed a high level of evidence of the pooled studies (SMD 1.02; 95%CI 0.22 to 2.27), according to the GRADE tool. This analysis revealed that our meta-analysis presented confidence in the estimated effect.

### 3.4 Sensitivity Analysis

The substantial I2 values presented in the analysis of FS decreased after removing one study at a time. For this, we have conducted tree analysis, in which the studies were removed as follows: for the sensitivity analysis 1, after the removal of the study performed by Wang *et al.* [60], because of the inclusion of only immunodeficient animals, the I2 dropped from 53% to 25%. For sensitivity analysis 2, after the removal of the study conducted by Dolinsk *et al.* [49], due to including animals that received a triple dosage of 8 mg·kg-1 in just one session per week, the I2 dropped from 53% to 44%. Regarding the sensitivity analysis of the LVEF, after the removal of the study performed by Yang *et al.* [61], due to dosage ~ 2142% higher than the dosage used as standard in the other studies polled in this meta-analysis, the I2 dropped from 82% to 16%.

## 4 DISCUSSION

The main outcome suggested that exercise offers cardioprotection when prescribed concomitantly with DOX in rodents, mainly in FS, the most frequently used index to assess ventricular mechanics (the percentage change in the diameter of the left ventricle during ventricular systole) [24, 25]. Cardiac function, measured by M-mode-derived changes in the left ventricular dimension, is most easily assessed by fractional shortening [63]. The normal value fluctuates between 28% and 44% (36% on average). This index is independent of age and heart rate, but it is dependent on pre and after-load [21, 22]. Although LVEF as an index of LV systolic function has broad applications, it also has important shortcomings. Two-dimensional LVEF estimations based on biplane Simpson’s method of discs suffer from modest reproducibility, as the exact imaging planes are difficult to recapture [64]. Even under optimal imaging conditions, LVEF can be an incomplete or even an incorrect estimate of LVEF function. This is especially true in significant concentric remodeling, hypertrophic cardiomyopathy, or small cavity size, where there is significant systolic dysfunction with reduced stroke volume despite a normal or supranormal LVEF [65].

### 4.1 Cardiotoxicity and DOX Dosage

Cumulative doses of DOX for cancer treatment in humans (ranging from 150, 250, 400, 550, and 700 mg·m2-1) report an incidence of heart failure in 5 to 48% of cases. Although there is no safe dose, higher cumulative doses carry a higher risk of heart failure [66].

The values for dose/treatment in humans must not exceed 14 mg·kg^-1^ [31]. Concerning these recommendations, we highlight some relevant considerations in the following studies. Hydock *et al.* [51] determined that daily DOX administration (1 mg·kg^-1^·day^-1^) for 15 days had a greater impact on diastolic function than a single weekly dose of DOX (2.5 mg·kg^-1^) for 6 weeks. On the other hand, Gomes-Santos *et al.* [50] verified that the exercise intervention did not attenuate cardiac dysfunction caused by DOX administration. Notably, the total dose of DOX was 25 mg·kg^-1^, 78% higher than the maximum recommended dose in humans. Likewise, Dolinsky *et al.* [49] used a total dose of DOX as 32 mg·kg^-1^, approximately 128% higher than the maximum recommended dose in humans partially, with the exercise group attenuating DOX-induced LV remodeling. Sturgeon *et al.* [52] demonstrated that despite the cardiac loss induced by DOX administration, the cardiac function determined by the echocardiogram was similar between the groups. This study had the lowest cumulative dose (4 mg·kg^-1^). Interestingly and differently from studies by Gomes-Santos *et al.* [50] and Dolinsky *et al.* [49], in the study by Yang *et al.* [61], rats received the highest dose of DOX injections (20 mg·kg^-1^ DOX, 3 times·week^-1^) for 5 weeks, and exhibited greatest impairment of heart rate in both FS and LVEF. Despite that, the exercise intervention restored cardiac function, with higher values of LVEF and FS compared to the DOX+SED group. Although Wang *et al.* [60] used 4-week-old young mice, they calculated the dose of DOX administration (13.8 mg·kg^-1^) from the Centers for Disease Control and Prevention (CDC) growth charts for a 5-year-old child based on the 50th percentile for height and weight.

The DOX accumulation inside the mitochondria has serious consequences for organelle function, including an increase in proton leakage into the mitochondrial matrix, which decreases the efficiency of mitochondrial energy production [67]. An exacerbated increase in reactive oxygen species and subsequent mitochondrial dysfunction appears to mediate cardiomyocyte apoptosis, leading to heart failure [39].

### 4.2 Exercise and Cardioprotection

The exercise program was reported to attenuate cardiac levels of Atrial Natriuretic Peptide (ANP), a gene that is expressed in response to increased cardiac afterload or cardiac injury, and prevent the reduction in the expression of calcium transporter ATP2A2 (ATPase sarcoplasmic/endoplasmic reticulum Ca^2+^ transporting 2) in the reticulum, being directly associated with the reduction of cardiomyocyte contractility [49, 51]. There was also an increase in the expression of mitofusin-1 and -2, responsible for mitochondrial fusion, and preservation of the levels of cardiac proteins of complexes I and II was also seen (*p <* 0.05) [21].

Exercise reduced DOX-induced oxidative stress after 2 weeks in Peripheral Blood Mononuclear Cells (PBMCs, an important marker of mitochondrial dysfunction) and improved cardiac fibrosis in both immunodeficient and immunocompetent mice [52]. It also reduced the expression of inflammatory mediators (IκBα: nuclear factor of kappa light polypeptide gene enhancer in B-cells inhibitor alpha, NF-κB: nuclear factor kappa B, COX-2: cyclooxygenase-2, and IL-8: interleukin-8), as well as fibrosis factors (TGF-β1: transforming growth factor beta-1, pERK: phosphorylated ERK, and CTGF: connective tissue growth factor), and cardiac remodeling factors (FGF2: fibroblast growth factor 2, uPA (serine protease urokinase plasminogen activator), and MMP-2 and MMP-9 (matrix metalloproteases), including two commonly used indicators of cardiac injury, lactate dehydrogenase, and creatine kinase-MB [61]. In agreement with the previous study, exercise intervention for cardiotoxicity induced by DOX promoted improvements in redox balance and protection of mitochondrial homeostasis since it helps to increase levels of the antioxidant enzymes Catalase (CAT), Glutamine Peroxidase (GP), Superoxide Dismutase (SOD), and Succinate Oxidase (SO) [68]. It also induces the action of molecular chaperones (HSPs), which play an important role in the protection against ischemia injury, reperfusion, and other stress stimuli [69]. Furthermore, physical exercise provides control over protein folding, prevention of denaturation, aggregation, and acceleration of the breakdown of damaged proteins [70]. It is associated with the preservation of cardiac function induced by oxidative stress states caused by the administration of DOX [71, 72] and reducing the level of drug penetration into cardiac tissue [60].

Regarding the exercise programs proposed by the studies included in our review, all of them used low/moderate intensity exercise programs (at ~40/60% of 〖”V” ˙”O” 〗_”2MAX”) according to the guidelines of the American College of Sports Medicine [62]. Two studies included freewheel for 24 hours [51, 58], and the others involved controlled speed on a treadmill, ranging from 10 m·min^-1^ to 18 m·min^-1^, lasting between 30 and 60 minutes. It should be noted that voluntary running on wheels is a model of physical activity, while the motorized treadmill (forced training) is a model of exercise [73]. The decision of which exercise modality to choose should depend on whether the investigators consider the results of the study to suggest protocols of use that corroborate the results, or just as mechanisms of clinical observation [74]. Factors, such as intensity, frequency, and duration, need to be better structured and confer remarkably different physiological, biochemical, and gene expression adaptations [75, 76]. However, a key question is whether these differences translate into differential effects on outcomes [77].

## 5 LIMITATIONS AND FUTURE RESEARCH

The way to determine cardiotoxicity is multifactorial in the spectrum related to oxidative stress and involves several markers. It is difficult to determine the degree of importance that each one has for the outcome of cardiotoxicity [78].

The most serious side effect of prolonged use of DOX is congestive heart failure [49]. Therefore, it is pertinent to mention that in the study model by Dolisnky *et al.* [49], the authors used mice that would not develop congestive heart failure. Hayward *et al.* [60] and Wang *et al.* [59, 60] used young 4-week-old mice, unlike other studies that have used adult animal models. In addition, Wang *et al.* [60] used Non-tumor-bearing Immunocompetent C57Bl/6J mice (NTB-IC) and mice bearing a palpable tumor (TB-NM) (Ewing sarcoma cells); the authors highlighted that tumor-bearing animals were more sensitive to DOX, while immunocompetent mice were much more resistant.

Cardiotoxicity is expressed as a reduction in LVEF (≥ 10%) below the lower limit of normal (<50%) [10, 77]. The usual standard for assessing heart failure is monitoring LVEF by cardiac imaging performed by two-dimensional echocardiography [79]. A recent meta-analysis involving breast cancer patients undergoing an exercise program demonstrated two-dimensional echocardiography to have limitations in the diagnosis of cardiotoxicity, especially subclinical cardiotoxicity [80]. However, the most widely used method in animal research studies was the assessment of FS, which was the primary outcome of our study. Furthermore, future studies may consider the effects of exercise concomitantly with DOX administration.

## CONCLUSION

This meta-analysis has verified that physical exercise exhibits a cardioprotective effect in rodents, as demonstrated by the evaluation of fractional shortening.

## Figures and Tables

**Table:** Search strategies details.

**Database**	**Search Phrase**
MedLine	(Exercise) OR (Physical Exercise) OR (Exercise Training) OR (Voluntary Exercise) OR (Resistance Exercise) OR (Aerobic Training) OR (Physical Training) OR (Interval Training) OR (Resistance Training) OR (Endurance) OR (Running) OR (Well Treadmill) OR (Aerobic Exercise) AND (Anthracycline) OR (Doxorubicin) OR (Taxanes) OR (Doxorubicin Hydrochloride) OR (Cyclophosphamide) OR (Epirubicin) OR (Gemcitabine) OR (Vinorelbine) AND (Cardiotoxicity) OR (Cardiotoxic) OR (Cardiac Function) OR (Cardiac Toxicity) OR (Cardiac Insufficiency) OR (Heart Failure) OR (Cardiac Dysfunction)
PubMed	(Exercise) OR (Physical Exercise) OR (Exercise Training) OR (Voluntary Exercise) OR (Resistance Exercise) OR (Aerobic Training) OR (Physical Training) OR (Interval Training) OR (Resistance Training) OR (Endurance) OR (Running) OR (Well Treadmill) OR (Aerobic Exercise) AND (Anthracycline) OR (Doxorubicin) OR (Taxanes) OR (Doxorubicin Hydrochloride) OR (Cyclophosphamide) OR (Epirubicin) OR (Gemcitabine) OR (Vinorelbine) AND (Cardiotoxicity) OR (Cardiotoxic) OR (Cardiac Function) OR (Cardiac Toxicity) OR (Cardiac Insufficiency) OR (Heart Failure) OR (Cardiac Dysfunction)
Web of Science	“All fields: (Doxorubicin) AND All fields: (Exercise). Overtime: All years.”
Scopus	“TITLE-ABS-KEY (Doxorubicin AND Exercise)”

**Fig. (2):**
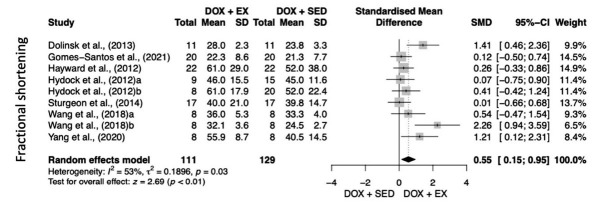
Fractional Shortening (FS) meta-analysis. meta-analysis. Total: number of studies included in the analysis, SMD: standard mean difference, 95%CI: confidence interval, I2: heterogeneity, Z (p): test for overall effect and *p*-value. *Statistical significance: *p* ≤ 0.05. **Abbreviations:** DOX + EX: Doxorubicin exercise intervention group; DOX + SED: Doxorubicin control group.

**Table 1:** Summary of sample and study characteristics.

**Study**	**Animals**	**Groups**	**Posology**	**Exercise Progression**	**Analysis**
Dolinsky *et al.* [48]	C57BL6 10 weeks females randomized	3 groups N = 9/11 SED + SAL SED + DOX DOX + EXE	8 mg·kg^-1^ 1 week^-1^ over 4 weeks	Acclimatization for 5 days on a motorized treadmill (10m·min^-1^) for 30 minutes. Training: 18 m·min^-1^ at 0% incline over 45 min; 5 days·week^-1^ over 8 weeks on a motorized treadmill	Transthoracic echocardiography 30 MHz transducer
Gomes-Santos *et al.* [49]	C57BL6 8 weeks Males randomized	3 groups SED + SAL N=12 SED + DOX N=20 DOX + EXE N=20	5 mg·kg^-1^ 1 week^-1^ over 5 weeks	40-50% of maximum exercise capacity at 0% grade, 40 min·session^-1^, 4 days·week^-1^ over 5 weeks	Transthoracic echocardiography transducer 40 MHz
Hayward *et al.* [57]	Male Sprague-Dawley rats 25 days (youth)	3 groups SED + SAL N=10 SED + DOX N=22 DOX + EXE N=22	DOX at 2 mg·kg^-1^on 7 consecutive days (cumulative dose 14 mg·kg^-1^)	Free access to racing wheels (MiniMitter, Bend, OR) 24 hours·day^-1^ over 10 weeks	Echocardiography
Hydock *et al.* [50]	Female Sprague-Dawley	6 groups SED + DOX N=24 SED + SAL N=16 WR + SAL N=8 WR + DOX N=9 SED + DOX N=20 SED + SAL N= 15 WR + SAL N= 8 WR + DOX N= 10	1 mg·kg^-1^ intraperitoneal DOX injections administered for 15 consecutive days (DOX) 2.5 mg·kg^-1^ intraperitoneal DOX injections administered over 6 weeks	Volunteer treadmill over 10 weeks	Transthoracic echocardiography transducer 10 MHz
Urgeon *et al.* [51]	C57BL/6 six to eight-week-old males	SED + SAL N=17 EXE + SAL N= 16 DOX + SED N= 17 DOX + EXE N=17	2 mg·kg^-1^ 1 x week^-1^ over 2 weeks	10 m·min^-1^ over 45 min, 5 days x week^-1^	Transthoracic echocardiography 30 MHz transducer
Wang *et al.* [58]	Nude or Balb/c mice deficient in T cells at four weeks of age and immunocompetent	N= 8 per group SED + SAL EXE + SAL DOX + SED DOX + EXE	2.5 mg·kg^-1^ 2 x week^-1^ over 2 weeks	45 minutes of treadmill walking x day^-1^, 5 x days week^-1^ over 12 weeks	The echocardiographic assessment was performed before and 24 h after therapy and then at 2, 4, 8, and 12 weeks after therapy
Wang *et al.* [59]	C57Bl/6J mice, 4 weeks old	N=8 per group SED + SAL EXE + SAL DOX + SED DOX + EXE	2.5 mg·kg^-1^ DOX 2 x week^-1^ over 2 weeks	Treadmill exercise 5 days x week^-1^, 45 minutes·day^-1^, 12 meters x min^-1^ over 2 weeks	Echocardiography was performed before the first exercise session in all mice and again on the last day of the experiment before euthanasia
Yang *et al.* [60]	A total of 24 male Sprague Dawley (SD) rats weighing 220 ± 20 g were used in this study (8 in each group)	N=8 per group SED + SAL SED + DOX DOX + EXE	20 mg·kg^-1^ of DOX was injected in the DOX group and the DOX+EXE group *via* intraperitoneal injection 3 x week^-1^	Exercise program (40 min x day^-1^ at 9 m x min^-1^ over 7 days) for familiarization with the treadmill. In the next step, the animals were trained with 60 min at 12 m·min^-1^ of treadmill exercise x day^-1^ over 28 days	Echocardiographic data were collected by a Vevo 770 microimaging system with a 25 MHz probe

**Abbreviations:** SAL: saline; SED: sedentary; DOX: doxorubicin; EXE: exercise; ×/week: times per week.

**Table 2:** Systematic review centre for laboratory animal experimentation (SYRCLE).

**Studies**	**1**	**2**	**3**	**4**	**5**	**6**	**7**	**8**	**9**	**10**
Dolinsky *et al.* [48]	U	Y	U	U	U	Y	Y	Y	U	U
Gomes-Santos *et al.* [49]	U	Y	U	U	U	U	U	N	U	U
Hayward *et al.* [57]	U	Y	N	U	N	U	N	U	U	U
Hydock *et al.* [50]	U	Y	N	U	N	U	U	U	U	U
Sturgeon *et al.* [51]	U	Y	U	U	U	U	U	N	U	U
Wang *et al.* [58]	U	Y	U	U	U	U	U	N	U	U
Wang *et al.* [59]	U	Y	U	U	U	U	U	N	U	U
Yang *et al.* [60]	U	Y	U	U	U	U	U	N	U	U

**Note: ** 1. Was the allocation sequence adequately generated and applied?; 2. Were the groups similar at baseline or were they adjusted for confounders in the analysis?; 3. Was the allocation to the different groups adequately concealed?; 4. Were the animals randomly housed during the experiment?; 5. Were the caregivers and/or investigators blinded from knowledge of which intervention each animal received during the experiment?; 6. Were animals selected at random for outcome assessment?; 7. Was the outcome assessor blinded?; 8. Were incomplete outcome data adequately addressed?; 9. Are reports of the study free of selective outcome reporting?; 10. Was the study free of other problems that could result in a high risk of bias?; Y, yes, low risk of bias; U, unclear, unclear risk of bias; N, no, high risk of bias.

**Table 3:** Collaborative approach to meta-analysis and review of animal data from experimental studies (CAMARADES).

**Studies**	**a**	**b**	**c**	**d**	**e**	**f**	**g**	**h**	**i**	**Total**
Dolinsky *et al.* [48]	1	1	1	1	1	0	1	1	1	8
Gomes-Santos *et al.* [49]	1	1	1	0	1	0	1	1	0	6
Hayward *et al.* [57]	1	1	1	0	1	0	1	1	1	7
Hydock *et al.* [50]	1	1	1	0	0	0	1	0	1	5
Sturgeon *et al.* [51]	1	1	1	0	1	0	1	1	0	6
Wang *et al.* [58]	1	1	1	1	1	0	1	1	1	8
Wang *et al.* [59]	1	1	1	1	1	0	1	1	1	8
Yang *et al.* [60]	1	1	1	1	1	0	1	1	1	8

**Note: ** 0 = no absence of information, high risk of bias; 1 = yes, information present, low risk of bias; a: Peer review publication; b: Presence of randomization; c: Assessment of the dose-response relationship; d: Blinded assessment of behavioral outcome; e: Monitoring of body weight parameters; f: Sample size calculation; g: Statement of compliance with regulatory requirements; h: Statement of potential conflicts of interest; i: Use of accurate/ suitable/ adequate dose to an animal model.

**Table 4:** Level of evidence (GRADE).

**Certainty Assessment**							**No. of** **Patients**		**Effect**		**Certainty**	**Importance**
**No. of Studies**	**Study Design**	**Risk of Bias**	**Inconsistency**	**Indirectness**	**Imprecision**	**Other** **Considerations**	**DOX + EXE**	**DOX + SED**	**Relative** **(95% CI)**	**Absolute** **(95% CI)**		
**Fractional Shortening (FS)**												
9	RCTs	not serious	not serious	not serious	not serious	none	111	129	–	SMD 0.55 SD higher (0.15 higher to 0.95 higher)	⨁⨁⨁⨁ High	Important
**Ejection Fraction (LVEF)**												
3	RCTs	not serious	not serious	not serious	not serious	none	39	39	–	SMD 1.03 SD higher (0.22 lower to 2.27 higher)	⨁⨁⨁⨁ High	Important

**Abbreviations:** SED + SAL (Sedentary + Saline), DOX + SED (Doxorubicin + Sedentary), EXE + SAL (Exercise + Saline), DOX + EXE (Doxorubicin + Exercise) RCTs: randomized controlled trials; CI: confidence interval; SMD: standard mean difference.

## Data Availability

All data generated or analyzed during this study are included in this published article.

## LIST OF ABBREVIATIONS

ANP: Atrial Natriuretic Peptide
ATP2A2: Sarcoplasmic ATPase/Endoplasmic Reticulum Ca^2+^ Transporting 2
CAT: Catalase
CDC: Centers for Disease Control
*COX*-*2*: Cyclooxygenase-2
*CTGF*: *= *Connective Tissue Growth Factor
DOX: Doxorubicin
FGF2: Fibroblast Growth Factor 2
FS: Fractional Shortening
GP: Glutamine Peroxidase
HSPS: Molecular Chaperones
IL-8: Interleukin-8
*IκBα*: Nuclear Factor of Kappa Light Polypeptide Gene Enhancer in B-Cells Inhibitor, Alpha
LVEF: Left Ventricular Ejection Fraction
MMP-2 and MMP-9: Matrix Metalloproteases
*NF*-*κB*: Factor Nuclear Kappa B
PBMCs: Peripheral Blood Mononuclear Cells
pERK: Phosphorylated ERK
SO: Succinate Oxidas
SOD: Superoxide Dismutase
TGF-β1: Transforming Growth Factor βeta-1
uPA: Serine Protease Urokinase Plasminogen Activator
VO_2MAX_: Maximal Oxygen Uptake

## STANDARDS OF REPORTING

PRISMA guidelines and methodologies were followed.

## SUPPLEMENTARY MATERIAL

PRISMA checklist is available as supplementary material on the publisher’s website along with the published article.
