# Supplementary material for: Effects of Physical Exercise and the use of Doxorubicin on Cardiac Function in Rodents: A Systematic Review and Meta-Analysis
Source: Curr Cardiol Rev. 2025 Feb 10;21(4):E1573403X328856. doi: 10.2174/011573403X328856241219114652 (PMC12180366; doi:10.2174/011573403X328856241219114652)
Supplement: Supplementary file 1 — PRISMA checklist is available as supplementary material on the publisher’s website along with the published article. [file CCR-21-4-E1573403X328856_SD1.pdf]

Supplementary Material

Effects of Physical Exercise and the Use of Doxorubicin on Cardiac Function in Rodents: A Systematic Review and Meta-Analysis

Bruno Gama Linhares[1], Diego Gama Linhares[2,3,*] and Rodrigo Gomes de Souza Vale[2,3]

[1]Research Center in Physical Activity, Health, and Leisure (CIAFEL), Faculty of Sport, University of Porto, Porto, Portugal; [2]Health Sciences Laboratory, Estácio de Sá University, Campos dos Goytacazes, Rio de Janeiro, Brazil; [3]Laboratory of Exercise and Sport, Institute of Physical Education and Sports, Rio de Janeiro State University, Rio de Janeiro, Brazil

Section and Topic	Item #	Checklist item	Location where item is reported
**TITLE**			
Title	1	Identify the report as a systematic review.	Line 3
**ABSTRACT**			
Abstract	2	See the PRISMA 2020 for Abstracts checklist.	Line 10-15
**INTRODUCTION**			
Rationale	3	Describe the rationale for the review in the context of existing knowledge.	Line 110-126
Objectives	4	Provide an explicit statement of the objective(s) or question(s) the review addresses.	Line 127-138
**METHODS**			
Eligibility criteria	5	Specify the inclusion and exclusion criteria for the review and how studies were grouped for the syntheses.	Line 148-175
Information sources	6	Specify all databases, registers, websites, organisations, reference lists and other sources searched or consulted to identify studies. Specify the date when each source was last searched or consulted.	Line 177-181
Search strategy	7	Present the full search strategies for all databases, registers and websites, including any filters and limits used.	Line 180-185
Selection process	8	Specify the methods used to decide whether a study met the inclusion criteria of the review, including how many reviewers screened each record and each report retrieved, whether they worked independently, and if applicable, details of automation tools used in the process.	Line 188-193
Data collection process	9	Specify the methods used to collect data from reports, including how many reviewers collected data from each report, whether they worked independently, any processes for obtaining or confirming data from study investigators, and if applicable, details of automation tools used in the process.	Line 188-193
Data items	10a	List and define all outcomes for which data were sought. Specify whether all results that were compatible with each outcome domain in each study were sought (e.g. for all measures, time points, analyses), and if not, the methods used to decide which results to collect.	Line 196-201
	10b	List and define all other variables for which data were sought (e.g. participant and intervention characteristics, funding sources). Describe any assumptions made about any missing or unclear information.	Line 319-330
Study risk of bias assessment	11	Specify the methods used to assess risk of bias in the included studies, including details of the tool(s) used, how many reviewers assessed each study and whether they worked independently, and if applicable, details of automation tools used in the process.	Line 209-224
Effect measures	12	Specify for each outcome the effect measure(s) (e.g. risk ratio, mean difference) used in the synthesis or presentation of results.	Line 238-246
Synthesis methods	13a	Describe the processes used to decide which studies were eligible for each synthesis (e.g. tabulating the study intervention characteristics and comparing against the planned groups for each synthesis (item #5)).	Line 201-207
	13b	Describe any methods required to prepare the data for presentation or synthesis, such as handling of missing summary statistics, or data conversions.	Line 201-207
	13c	Describe any methods used to tabulate or visually display results of individual studies and syntheses.	Table 2
	13d	Describe any methods used to synthesize results and provide a rationale for the choice(s). If meta-analysis was performed, describe the model(s), method(s) to identify the presence and extent of statistical heterogeneity, and software package(s) used.	Line 299
	13e	Describe any methods used to explore possible causes of heterogeneity among study results (e.g. subgroup analysis, meta-regression).	Not applicable
	13f	Describe any sensitivity analyses conducted to assess robustness of the synthesized results.	Line 320-331
Reporting bias assessment	14	Describe any methods used to assess risk of bias due to missing results in a synthesis (arising from reporting biases).	Line 282 -296
Certainty assessment	15	Describe any methods used to assess certainty (or confidence) in the body of evidence for an outcome.	Line 309 - 317

Section and Topic	Item #	Checklist item	Location where item is reported
**RESULTS**			
Study selection	16a	Describe the results of the search and selection process, from the number of records identified in the search to the number of studies included in the review, ideally using a flow diagram.	Line 249- 255
	16b	Cite studies that might appear to meet the inclusion criteria, but which were excluded, and explain why they were excluded.	Line 257-268
Study characteris-tics	17	Cite each included study and present its characteristics.	Table 2
Risk of bias in studies	18	Present assessments of risk of bias for each included study.	Table 3-4
Results of indivi-dual studies	19	For all outcomes, present, for each study: (a) summary statistics for each group (where appropriate) and (b) an effect estimate and its precision (e.g. confidence/credible interval), ideally using structured tables or plots.	Figure 2 and 3
Results of synt-heses	20a	For each synthesis, briefly summarise the characteristics and risk of bias among contributing studies.	Line 207-222
	20b	Present results of all statistical syntheses conducted. If meta-analysis was done, present for each the sum-mary estimate and its precision (e.g. confidence/credible interval) and measures of statistical heterogeneity. If comparing groups, describe the direction of the effect.	Figure 2 and 3
	20c	Present results of all investigations of possible causes of heterogeneity among study results.	Line 319 - 330
	20d	Present results of all sensitivity analyses conducted to assess the robustness of the synthesized results.	Line 319 - 330
Reporting biases	21	Present assessments of risk of bias due to missing results (arising from reporting biases) for each synthesis assessed.	Table 3-4
Certainty of evi-dence	22	Present assessments of certainty (or confidence) in the body of evidence for each outcome assessed.	Line 309-315
**DISCUSSION**			
Discussion	23a	Provide a general interpretation of the results in the context of other evidence.	Line 334-341
	23b	Discuss any limitations of the evidence included in the review.	Line 346-369
	23c	Discuss any limitations of the review processes used.	Line 432-458
	23d	Discuss implications of the results for practice, policy, and future research.	Line 458-463
**OTHER INFORMATION**			
Registration and protocol	24a	Provide registration information for the review, including register name and registration number, or state that the review was not registered.	Line 139-144
	24b	Indicate where the review protocol can be accessed, or state that a protocol was not prepared.	Not applicable
	24c	Describe and explain any amendments to information provided at registration or in the protocol.	Not applicable
Support	25	Describe sources of financial or non-financial support for the review, and the role of the funders or sponsors in the review.	Line 493 - 495
Competing interests	26	Declare any competing interests of review authors.	Line 497-550
Availability of data, code and other materials	27	Report which of the following are publicly available and where they can be found: template data collection forms; data extracted from included studies; data used for all analyses; analytic code; any other materials used in the review.	Not applicable

From: Page MJ, McKenzie JE, Bossuyt PM, Boutron I, Hoffmann TC, Mulrow CD, et al. The PRISMA 2020 statement: an updated guideline for reporting systematic reviews. BMJ 2021;372:n71. doi: 10.1136/bmj.n71

For more information, visit: http://www.prisma-statement.org/
